# Abnormal islet sphingolipid metabolism in type 1 diabetes

**DOI:** 10.1007/s00125-018-4614-2

**Published:** 2018-04-18

**Authors:** Laurits J. Holm, Lars Krogvold, Jane P. Hasselby, Simranjeet Kaur, Laura A. Claessens, Mark A. Russell, Clayton E. Mathews, Kristian F. Hanssen, Noel G. Morgan, Bobby P. C. Koeleman, Bart O. Roep, Ivan C. Gerling, Flemming Pociot, Knut Dahl-Jørgensen, Karsten Buschard

**Affiliations:** 1 The Bartholin Institute, Department of Pathology, Rigshospitalet, Copenhagen Biocenter, Ole Maaløes Vej 5, 2200 Copenhagen N, Denmark; 2 Division of Paediatric and Adolescent Medicine, Oslo University Hospital, Oslo, Norway; 3 Faculty of Odontology, University of Oslo, Oslo, Norway; 4 Department of Pathology, Rigshospitalet, Copenhagen, Denmark; 5 Steno Diabetes Center Copenhagen, Gentofte, Denmark; 6 Department of Immunohaematology & Blood Transfusion, Leiden University Medical Center, Leiden, the Netherlands; 7 Department of Medical Genetics, University Medical Center, Utrecht, the Netherlands; 8 Institute of Biomedical and Clinical Sciences, University of Exeter Medical School, Exeter, UK; 9 Department of Pathology, University of Florida, Gainesville, FL USA; 10 Department of Endocrinology, Oslo University Hospital, Oslo, Norway; 11 Department of Diabetes Immunology, Diabetes & Metabolism Research Institute, Beckman Research Institute at the City of Hope, Duarte, CA USA; 12 Department of Medicine, University of Tennessee, Memphis, TN USA; 13 Faculty of Medicine, University of Oslo, Oslo, Norway

**Keywords:** Fenofibrate, Gene polymorphisms, GWAS, Islet autoimmunity, NOD mice, Prevention, Sphingolipid, Sulfatide, T cells, Type 1 diabetes

## Abstract

**Aims/hypothesis:**

Sphingolipids play important roles in beta cell physiology, by regulating proinsulin folding and insulin secretion and in controlling apoptosis, as studied in animal models and cell cultures. Here we investigate whether sphingolipid metabolism may contribute to the pathogenesis of human type 1 diabetes and whether increasing the levels of the sphingolipid sulfatide would prevent models of diabetes in NOD mice.

**Methods:**

We examined the amount and distribution of sulfatide in human pancreatic islets by immunohistochemistry, immunofluorescence and electron microscopy. Transcriptional analysis was used to evaluate expression of sphingolipid-related genes in isolated human islets. Genome-wide association studies (GWAS) and a T cell proliferation assay were used to identify type 1 diabetes related polymorphisms and test how these affect cellular islet autoimmunity. Finally, we treated NOD mice with fenofibrate, a known activator of sulfatide biosynthesis, to evaluate the effect on experimental autoimmune diabetes development.

**Results:**

We found reduced amounts of sulfatide, 23% of the levels in control participants, in pancreatic islets of individuals with newly diagnosed type 1 diabetes, which were associated with reduced expression of enzymes involved in sphingolipid metabolism. Next, we discovered eight gene polymorphisms (*ORMDL3*, *SPHK2*, *B4GALNT1*, *SLC1A5*, *GALC*, *PPARD*, *PPARG* and *B4GALT1*) involved in sphingolipid metabolism that contribute to the genetic predisposition to type 1 diabetes. These gene polymorphisms correlated with the degree of cellular islet autoimmunity in a cohort of individuals with type 1 diabetes. Finally, using fenofibrate, which activates sulfatide biosynthesis, we completely prevented diabetes in NOD mice and even reversed the disease in half of otherwise diabetic animals.

**Conclusions/interpretation:**

These results indicate that islet sphingolipid metabolism is abnormal in type 1 diabetes and suggest that modulation may represent a novel therapeutic approach.

**Data availability:**

The RNA expression data is available online at https://www.dropbox.com/s/93mk5tzl5fdyo6b/Abnormal%20islet%20sphingolipid%20metabolism%20in%20type%201%20diabetes%2C%20RNA%20expression.xlsx?dl=0. A list of SNPs identified is available at https://www.dropbox.com/s/yfojma9xanpp2ju/Abnormal%20islet%20sphingolipid%20metabolism%20in%20type%201%20diabetes%20SNP.xlsx?dl=0.

**Electronic supplementary material:**

The online version of this article (10.1007/s00125-018-4614-2) contains peer-reviewed but unedited supplementary material, which is available to authorised users.

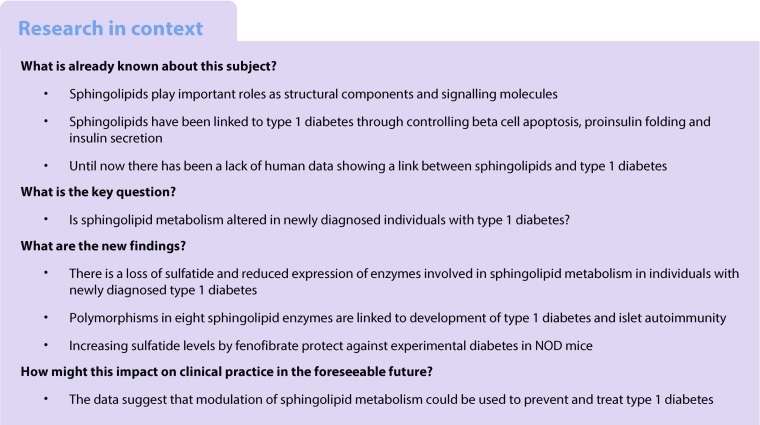



## Introduction

Type 1 diabetes is characterised as an autoimmune disease in which autoreactive T cells infiltrate the pancreatic islets and destroy the insulin producing beta cells [1]. However, the potential importance of beta cell dysfunction, rather than complete beta cell loss, in the pathogenesis of type 1 diabetes has recently been emphasised by the demonstration that a majority of individuals with diabetes retain a significant proportion of insulin-positive islets at disease onset [2–4]. In support of this, islets isolated from pancreatic biopsies taken from individuals with type 1 diabetes partly regained their ability to secrete insulin in response to glucose when cultured in a non-diabetogenic environment in vitro [5], while the majority of individuals with type 1 diabetes regained insulin production immediately upon treatment with autologous system cell therapy [6]. Potential key players in this beta cell dysfunction are sphingolipids, a diverse group of lipids found in all cellular membranes, having diverse roles as both structural components and signalling molecules [7–9]. Sphingolipids are known to have important roles in beta cell biology [10, 11] and have been linked to the development of diabetes-associated pathologies [12]. One especially important sphingolipid is sulfatide (3-O-sulfogalactosylceramide) which acts as an insulin chaperone, preserves insulin crystals and regulates insulin secretion by influencing the gating of ATP-sensitive potassium channels [13, 14]. However, most studies regarding the role of sphingolipid metabolism in beta cells have been conducted in animal models and cell cultures. Here, we tested the hypothesis that sphingolipid metabolism is contributing to the pathogenesis of human type 1 diabetes from several perspectives. Using blood and tissue samples from pancreas biopsies from newly diagnosed individuals with type 1 diabetes, we found evidence suggesting that sphingolipid metabolism plays a role in type 1 diabetes pathology. Based on this we tested whether increasing pancreatic sulfatide levels could prevent and reverse experimental autoimmune diabetes in NOD mice.

## Methods

### Human tissue

Pancreatic tissue was collected in the Diabetes Virus Detection (DiViD) studies as described previously [15]. In short, individuals with diabetes between 25 and 35 years of age had a surgical minimal pancreatic tail resection obtained by laparoscopy 3 to 9 weeks after the onset of type 1 diabetes. The DiViD study was approved by The Norwegian Government’s Regional Ethics Committee (reference 2009/1907). UK tissue samples from the Exeter Archival Diabetes Biobank had all been collected prior to the study and were made available with ethical approval from the West of Scotland Research Ethics Service (reference 15/WS/0258). The tissue from donors with type 2 diabetes and non-diabetic control donors, used for RNA analyses, was acquired from the network of Pancreatic Organ Donors (nPOD; with approval by the University of Tennessee Health Science Center (UTHSC) local Institutional Review Board [reference 10-00848-XM]). The pancreatic tissue from participants without diabetes used for sulfatide staining was acquired from Rigshospitalet, Copenhagen, Denmark, as completely anonymised (unknown age and sex) healthy tissue removed from pancreas samples after resection for surgical treatment of pancreatic cancer and was used in accordance with the rules by Region Hovedstaden Committee on Health Research Ethics. Human islets for electron microscopy were obtained as described previously [16]. Peripheral blood samples were collected from individuals with type 1 diabetes, aged between 1 and 39 years after informed consent was obtained as approved by the Medical Ethical Committee of Leiden University Medical Center (reference CME05/68C). For all participant information see electronic supplementary material (ESM) Table 1.

### Immunohistochemistry

Immunohistochemistry on neighbouring pancreatic sections from the DiViD study and control participants was performed using anti-sulfatide antibody Sulph I (a gift from P. Fredman, Gothenburg University, Sweden [17]; diluted 1:150) or guinea pig anti-insulin (Dako, Ely, UK; diluted 1:700). Visualisation was performed using ultraView Universal DAB Detection Kit (Roche, Basel, Switzerland). The light microscope BX51 (Olympus America, Melville, NY, USA) was used to analyse the stained specimens. For the UK sections, immunohistochemistry was visualised using Dako REAL EnVision Detection System, Peroxidase/DAB+ (Dako) and light microscope Nikon 50i Eclipse (Nikon, Kingston-upon-Thames, UK). The relative sulfatide level in pancreatic islets was compared with control participants without diabetes. Staining in all control participants was set to one (100%) and the staining intensity in minimum 30 islets from each donor was evaluated.

### Immunofluorescence staining

Pancreatic sections were stained with an anti-sulfatide antibody (diluted 1:150) and a secondary Alexa Fluor 488 antibody (Life Technologies, Paisley, UK; diluted 1:400). Pancreatic sections were co-stained with an anti-glucagon antibody raised in rabbit (Abcam, Cambridge, UK; diluted 1:4000) and with guinea pig anti-insulin (Dako; diluted 1:700) plus relevant secondary antibodies labelled with Alexa Fluor 647 and Alexa Fluor 568 (Life Technologies; (diluted 1:400). Images were captured under fluorescence illumination using a Leica AF6000 microscope (Leica, Milton Keynes, UK). Leica application suite X software (Lecia) was used to remove background staining and crop images.

### Electron microscopy

Isolated pancreatic human islets were incubated overnight at 4°C with Sulph I (diluted 1:1000) and washed in 1% PBS-BSA. Next the islets were incubated overnight at 4°C with 1 nm gold labelled goat anti-mouse IgG (Aurion, Wageningen, the Netherlands; diluted as 1:300). The islets were postfixed in 2% glutaraldehyde for 2 h, before silver enhancement using AURION R-GENT SE-EM (Aurion). The islets were then washed in distilled water before osmication in 1% OsO4 diluted in 0.1 mol/l cacodylate buffer. After washing in 0.1 mol/l cacodylate buffer, the specimens were dehydrated in alcohol and embedded in Epon Resin 812 before ultra-sections were examined in a Philips 208 electron microscope (Philips, Eindhoven, the Netherlands).

### RNA analyses

Frozen tissue (optimal cutting temperature [OCT]) sections were obtained from the nPOD [18] and DiViD tissue collections. Tissue slides were fixed and laser-capture of islets conducted as previously described [19]. All islets in two to five sections of tissue from each donor were captured and pooled and RNA extracted using the Arcturus PicoPure RNA Isolation Kit (Applied Biosystems, Grand Island, NY, USA). Quality and quantity of RNA was determined on a Bioanalyzer 2100 instrument (Agilent Technologies, Santa Clara, CA, USA). Samples with sufficient quantity and quality of RNA were then subjected to gene expression analysis using Affymetrix expression arrays (GeneChip Human Gene 2.0 ST) and scaled normalised gene expression values produced as previously described [20]. The normalised expression data for 70 genes of interest were then subjected to analysis as described below.

### GWAS analyses

The 70 genes examined at the RNA level were also evaluated using GWAS to look for a genetic association with type 1 diabetes. Immunochip SNPs for type 1 diabetes were retrieved from Onengut-Gumuscu et al 2015 [21]. A cut-off *p* value <0.02 was used to retrieve nominally significant SNPs. SNPs within (±100 kb flanking regions) of the examined genes were identified for further analysis. We used Encyclopedia of DNA Elements (ENCODE) regulatory features (ChIP-Seq peaks, DNase I hypersensitivity peaks, DNase I footprints) from University of California Santa Cruz (UCSC) genome browser [22] (http://genome.ucsc.edu/) and RegulomeDB [23] to identify potential regulatory SNPs likely to affect the expression of the associated gene. We also integrated data from multiple expression quantitative trait loci (eQTL) studies [24–26] to identify SNPs associated with changes in expression (*cis*-eQTLs) of their associated gene. The *cis*-eQTL effects were calculated using linear regression models in the selected tissues (whole blood, fibroblast and lymphoblastoid cell lines) using a *cis* window of ±1 MB around the transcription start site at significance level of *p* < 0.05 [24–26]. SNPs mapping to HLA regions were excluded from the analysis.

### Blood donors

Peripheral blood was collected from 71 individuals with type 1 diabetes. Peripheral blood mononuclear cells (PBMCs) were isolated by Ficoll density gradient centrifugation and resuspended in Iscove’s Modified Dulbecco’s Media (IMDM) (Life Technologies, Paisley, UK.) containing 10% heat-inactivated human serum (HS; Sanquin, the Netherlands). PBMCs were subsequently tested for the presence of autoreactive T cells using a T cell proliferation assay.

### SNP genotyping and genetic risk score

DNA was isolated from PBMCs of individuals with type 1 diabetes using the DNeasy Blood & Tissue Kit (Qiagen Benelux, Venlo, the Netherlands). DNA concentration was determined by NanoDrop and samples were concentrated at 50 ng/μl. SNP genotyping was performed on the Infinium ImmunoArray-24 v2 BeadChip Kit (Illumina, Eindhoven, the Netherlands) according to the manufacturer’s protocol.

To test the cumulative effect of identified SNPs on islet autoimmunity, we computed a genetic risk score (GRS) in all individuals with type 1 diabetes. GRS is the sum of the number of risk alleles (0, 1 or 2) multiplied by the natural log of the OR for each SNP, divided by the total number of SNPs. SNPs were also individually analysed. The SNPs selected for the study were based on the SNP with the lowest *p* value and the highest OR was examined for each of the genes identified in the GWAS analysis.

### T cell proliferation assay

A T cell proliferation assay was performed on PBMCs freshly isolated from individuals with type 1 diabetes to investigate autoimmunity towards GAD65, preproinsulin (PPI), islet antigen-2 (*IA*-*2*) and insulin-defective ribosomal product (INS-DRiP). Human recombinant proteins GAD65, PPI, IA-2 and INS-DRiP were produced as previously described [27, 28]. PBMCs were seeded (150,000/well) in round-bottomed 96-well microculture plates (Greiner, Nürtingen, Germany) and cultured for 5 days in IMDM containing 10% HS at 37°C in 5% CO_2_, in a humidified atmosphere. Cells were cultured in triplicates in medium alone, with 10 μg/ml recombinant GAD65, PPI, IA-2 or INS-DRiP or with recombinant IL-2 (35 units/mL; Genzyme, Cambridge, MA, USA) as positive control. After 16 h of culture, 50 μl RPMI medium 1640 (Dutch modification; Gibco, Thermo Fisher Scientific, Waltham, MA, USA) containing 18500 Bq [^3^H]thymidine (DuPont, Boston, MA, USA) was added per well. After the cells were harvested on filters with an automated harvester, proliferation was determined by the measurement of 3H-thymidine incorporation in an automatic liquid scintillation counter. All results are calculated as mean counts per min (CPM) in the presence of antigen and compared with medium alone. Stimulation index (SI) = mean CPM_ANTIGEN_/mean CPM_MEDIUM_. An SI ≥3 is considered positive. In three participants, INS-DRiP was not measured.

### Animals and diabetes monitoring

Female NOD mice (Taconic Biosciences, Hudson, NY, USA) were kept in a specific pathogen-free (SPF) animal facility (temperature 22°C, 12 h light cycle, air change 16 times per h and humidity 55 ± 10%). Animal experiments were approved by the Animal Experiments Inspectorate, Ministry of Food, Agriculture and Fisheries of Denmark (reference 2012-15-2934-00086) and experiments performed according to international guidelines for the care and use of laboratory animals. The mice had free access to drinking water and standard Altromin 1320 diet (Altromin, Lage, Germany) with or without 0.01% fenofibrate (Sigma, St Louis, MO, USA). The mice were inspected weekly for diabetes from an age of 84 days using FreeStyle Lite (Abbott, Chicago, IL, USA) glucose monitoring. Diabetes diagnosis was based on two blood glucose measurements >12 mmol/l with an interval of 2 days, all measurements were made between 09:00 and 13:00 hours. The date of the first blood glucose measurement >12 mmol/l was used as diabetes onset date. Mice were killed by cervical dislocation at onset of diabetes or at the age of 217 days. Distribution of animals into groups and diabetes monitoring was not performed blinded. In the reversal studies, fenofibrate treatment was immediately commenced at the onset of diabetes and continued for 3 weeks. No inclusion or exclusion criteria were used.

### Insulitis and sulfatide scoring of NOD mice

Insulitis score was calculated from six mice in each group, at 13 weeks old. Pancreases were removed, fixed in 10% neutral buffered formalin overnight, embedded in paraffin and sectioned in 5 μm sections that were subsequently stained in haematoxylin and eosin. The sections were evaluated randomly and blinded using an Olympus BX53 microscope (Olympus America). Twenty-five islets from each mouse were scored according to the following scale: 0, no infiltration; 1, intact islets but with few mononuclear cells surrounding the islets; 2, peri-insulitis; 3, islet infiltration below 50% and 4, islet infiltration above 50%. Neighbouring slides were stained for sulfatide and scanned using NanoZoomer-XR (Hamamatsu, Hamamatsu City, Japan). For each mouse one slide was scored and the staining intensity was evaluated using a scale from 0 to 4 with 0 denoting no sulfatide and 4 denoting intensity as seen in neurons. The scoring was performed blinded.

### Statistics

The statistical analysis was performed using GraphPad Prism version 6.01 (GraphPad, La Jolla, CA, USA) and data is shown as mean ± SEM unless otherwise noted. The cumulative diabetes incidence was assessed using logrank Mantel–Cox. Correlation between insulitis and sulfatide was performed with a linear regression. For comparisons between groups a two-tailed unpaired Student’s *t* test or a one-way ANOVA with Tukey’s multiple comparisons test. The percentage of participants with a positive T cell response was evaluated using a *χ*^2^ test and two-proportions *Z* test. Data were natural log-transformed before analysis if not normally distributed. A *p* value of less than 0.05 was considered significant. *p < 0.05; **p < 0.01; ***p < 0.001; ****p < 0.0001.

## Results

### Sulfatide is reduced in human pancreatic islets at the onset of type 1 diabetes

**Fig. 1:**
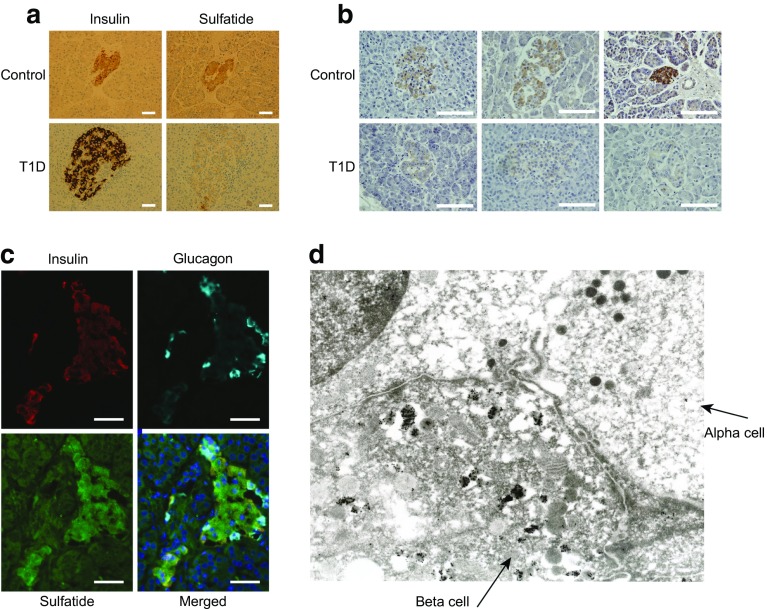
Sulfatide is present in beta cells and is lost from insulin-positive islets at the onset of type 1 diabetes. (**a**) Immunohistochemical staining of insulin and sulfatide from a control participant without diabetes and a patient with new-onset type 1 diabetes from DiViD. The pictures represent a standard islet as found in all six DiViD cases. Immunohistochemistry shows pronounced insulin staining and no sulfatide in individuals with type 1 diabetes. Scale bar, 50 μm. (**b**) Immunohistochemical staining of sulfatide of three healthy control participants and three newly diagnosed individuals with type 1 diabetes from the UK. Scale bar, 50 μm. (**c**) Immunofluorescent staining of a pancreas without diabetes showing that sulfatide is expressed in beta cells. Scale bar, 30 μm. (**d**) Electron microscopy on an isolated pancreatic human islet stained for sulfatide. Sulfatide is localised to insulin granules in beta cells. T1D, type 1 diabetes

To study sulfatide levels in human islets at the onset of type 1 diabetes, a sulfatide specific antibody was employed to compare islet immunostaining. Pancreas biopsies from individuals with newly diagnosed type 1 diabetes included in the DiViD study, had reduced sulfatide staining as compared with control participants without diabetes (Fig. 1a). Of the islets in the six DiViD individuals with diabetes, 63% were sulfatide positive and had a relative sulfatide staining intensity of 23% SEM ±6% (*p* < 0.0001) compared with the control group. This loss of sulfatide was observed in all six individuals studied and was confirmed in a separate cohort of individuals with type 1 diabetes from the Exeter Archival Diabetes Biobank (Fig. 1b).

Analysis of multiple islets from four pancreases revealed that sulfatide was found only in beta cells, but absent in alpha cells (Fig. 1c), which was confirmed by electron microscopy (Fig. 1d). Sulfatide was occasionally detectable in islet cells negatively for both insulin and glucagon (ESM Fig. 1).

### Reduced expression of enzymes involved in sphingolipid metabolism in human pancreatic islets at the onset of type 1 diabetes

**Fig. 2:**
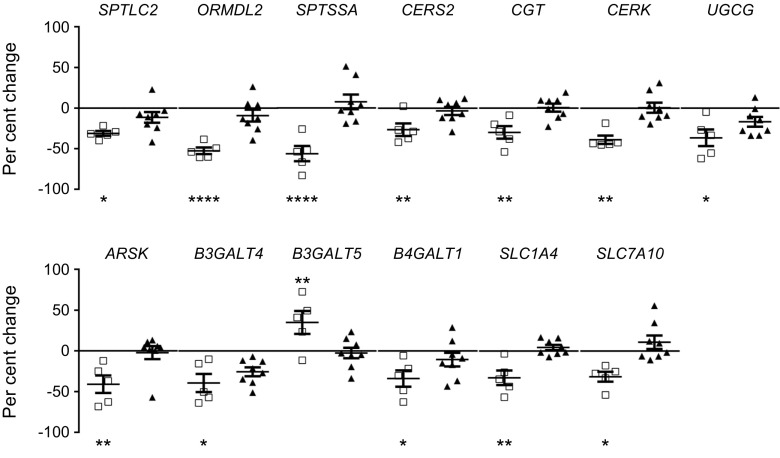
Altered expression of enzymes involved in sphingolipid metabolism at the onset of type 1 diabetes**.** RNA was isolated from laser-dissected islets and analysed by microarray. The individuals with type 1 diabetes are from the DiViD study. The mean difference in per cent ± SEM is shown. Solid line at zero represents control average. Control (*n*=18); white squares, type 1 diabetes (*n*=5); black triangles, type 2 diabetes (*n*=8). *p<0.05; **p<0.01; ****p<0.0001. One-way ANOVA with Tukey’s multiple comparisons test

A microarray analysis was performed to examine the expression of enzymes involved in sphingolipid metabolism in human islets. RNA was isolated from the islets of individuals with new onset type 1 diabetes (DiViD), individuals with type 2 diabetes and control participants without diabetes. RNA levels of 70 genes involved in sphingolipid metabolism were evaluated and 13 genes were found to have significantly altered expression at the onset of type 1 diabetes compared with control participants (Fig. 2). *SPTLC2* (which encodes serine palmitoyltransferase long chain base subunit 2), a subunit of serine palmitoyltransferase (SPT) which catalyses the first step in sphingolipid synthesis [29], was reduced (by 31%, *p* = 0.04). Similarly, the expression of the SPT inhibitor *ORMDL2* (encoding ORMDL sphingolipid biosynthesis regulator 2) [30] and activator *SPTSSA* (encoding serine palmitoyltransferase small subunit A) [31] were also reduced 53%, *p* < 0.0001; 56%, *p* < 0.0001, respectively. Expression of enzymes involved in the generation and modification of ceramide were also reduced, including ceramide synthase 2 (*CERS2*) which was decreased (26%, *p* = 0.004). Expression of ceramide galactosyltransferase (*CGT*, also known as *UGT8*), ceramide kinase (*CERK*) and ceramide glucosyltransferase (*UGCG*) were similarly diminished; by 30%, *p* = 0.005; 39%, *p* = 0.003 and 37%, *p* = 0.03, respectively. We also found a reduced expression of lysosomal arylsulfatase K (*ARSK*; 41%, *p* = 0.004). Our results indicate an altered distribution of complex glycosphingolipids in type 1 diabetes, with increased expression of the galactosyltransferase, *B3GALT5* (which encodes β-1,3-galactosyltransferase 5) (35%, *p* = 0.002), whereas *B3GALT4* (encoding β-1,3-galactosyltransferase 4) and *B4GALT1* (β-1,4-galactosyltransferase 1) were downregulated (by 39%, *p* = 0.01 and 34%, *p* = 0.02, respectively). Finally, we observed a reduced expression of two amino acid transporters; *SLC1A4* (encoding solute carrier family 1 member 4) (33%, *p* = 0.009) and *SLC7A10* (solute carrier family 7 member 10) (32%, *p* = 0.01) which facilitate the uptake of the sphingolipid precursor serine into cells [32]. None of the examined genes had altered expression in type 2 diabetes compared with control participants.

### SNPs in promoter regions of enzymes involved in sphingolipid metabolism associate with the development of type 1 diabetes

Next, we tested whether SNPs in genes involved in sphingolipid metabolism were associated with genetic predisposition to type 1 diabetes. GWAS have identified around 50 loci that influence the risk of developing type 1 diabetes but the disease-promoting genes at these loci often remain unknown [33]. We examined regions 100 kb upstream and downstream of the transcriptional start site of 70 genes involved in sphingolipid metabolism (Table 1). A *p* value <0.02 was selected to identify all SNPs associated with type 1 diabetes, as previously described [34]. SNPs mapping to the HLA regions were excluded from the analysis. RegulomeDB, which ranks SNPs based on the likelihood of the SNP influencing gene expression, was used with a cut-off of ≤3 to prioritise SNPs with a likely regulatory activity [23]. We identified eight genes with SNPs associated with an increased risk of type 1 diabetes and a RegulomeDB score ≤3 (Table 1) and OR up to 1.47. Five genes coding for enzymes involved in sphingolipid biosynthesis (*ORMDL3* [ORMDL sphingolipid biosynthesis regulator 3], *SPHK2* [sphingosine kinase 2], *B4GALNT1* [β-1,4-*N*-acetyl-galactosaminyltransferase 1], *GALC* [galactosylceramidase] and *B4GALT1*), two transcription factors (peroxisome proliferator-activated receptor (*PPARs*) *D* and *G*), which regulate the expression of enzymes in sphingolipid metabolism [35] and the amino acid transporter *SLC1A5* (solute carrier family 1 member 5), which is involved in the uptake of sphingolipid precursor l-serine (Table 1). Of these *ORMDL3* has been previously described [36]. Next, we used *cis*-eQTLs to evaluate the predicted effect of the SNPs on the expression levels of their associated gene. We integrated data from pre-calculated *cis*-eQTLs [24]. For six genes (*ORMDL3*, *GALC*, *SPHK2*, *SLC1A5*, *PPARD* and *B4GALT1*) the SNPs with the strongest association with type 1 diabetes (lowest *p* value) also acted as *cis*-eQTLs, suggesting that these SNPs regulate the expression of their associated genes (Table 1).

**Table 1:** Genes related to sphingolipid metabolism are in type 1 diabetes-associated genetic regions

Gene	Total type 1 diabetes SNPs (*p*<0.02)	SNPs (RegulomeDB ≤3)	OR (RegulomeDB ≤3)		*p* value (RegulomeDB ≤3)		*cis*-eQTL *p* value (tissue/cell-line)
*ORMDL3*	​155	​36	​1.20	(rs75290103)	1.20×10^−8^	(rs12150079)	2.6×10^−11^ (whole blood)
*SPHK2*	​82	​18	​1.13	(rs281388)	5.28×10^−10^	(rs33988101)	​0.032 (whole blood); 8.4×10^−4^ (cells: transformed fibroblasts)
*B4GALNT1*	​54	​14	​1.47	(rs41292013)	​9.43×10^−5^	(rs775251)	​​–
*SLC1A5*	​50	​14	​1.16	(rs10412340)	​4.72×10^−8^	(rs402072)	​1.4×10^−7^ (cells: transformed fibroblasts)
*GALC*	​42	​3	​1.06	(rs17798191)	​0.01	(rs10139328)	​2.3×10^−5^ (cells: EBV-transformed lymphocytes); 0.033 (whole blood)
*PPARD*	​27	​4	1.13	(rs7744392)	8.74×10^−3^	(rs774439​2)	​2.7×10^−3^ (cells: transformed fibroblasts)
*PPARG*	​4	​1	​1.17	(rs77040839)	​0.018	(rs77040839)	​–
*B4GALT1*	​1	​1	​1.12	(rs7019909)	​5.57×10^−4^	(rs7019909)	​7.7×10^−5^ (cells: transformed fibroblasts); 0.008 (cells: EBV-transformed lymphocytes)

Type 1 diabetes-associated SNPs (*p* < 0.02) were identified in eight genes (±100 kb) involved in sphingolipid metabolism. Genes located in proximity to HLA regions have been excluded. Genes are ranked according to the total number of type 1 diabetes-associated SNPs. The RegulomeDB score, which ranks SNPs based on the likelihood of the SNP influencing gene transcription (the lower the more likely), was used with a cut-off ≤3 to prioritise SNPs with a likely regulatory function. In genes with more than one SNP, the highest OR/lowest *p* value is mentioned. OR is based on the minor allele for *B4GALNT1*, *PPARG* and *SLC1A5* and the major allele for *B4GALT1*, *GALC*, *ORMDL3*, *PPARD* and *SPHK2*. The last column reports the *cis*-eQTL *p* value in disease relevant tissues/cell lines for the SNP with the lowest *p* value

### Increased genetic risk defined by sphingolipid-related SNPs is associated with reduced proliferation of islet-specific T cells in individuals with type 1 diabetes

We wanted to evaluate whether the identified SNPs could affect islet autoimmunity. The most promising SNPs (lowest *p* value and highest OR for each gene, as shown in Table 1) were selected and a GRS based on the number of risk alleles and OR per SNP was computed. The GRS was correlated to proliferation of T cells in response to islet autoantigens GAD65, PPI, IA-2 and INS-DRiP [28] as measured by the SI. A cohort of 71 individuals with type 1 diabetes were divided between three risk groups: low genetic risk (GRS = 0.11–0.14, *n* = 20); intermediate (GRS = 0.14–0.16, *n* = 37) and high (GRS > 0.16, *n* = 14).

**Fig. 3:**
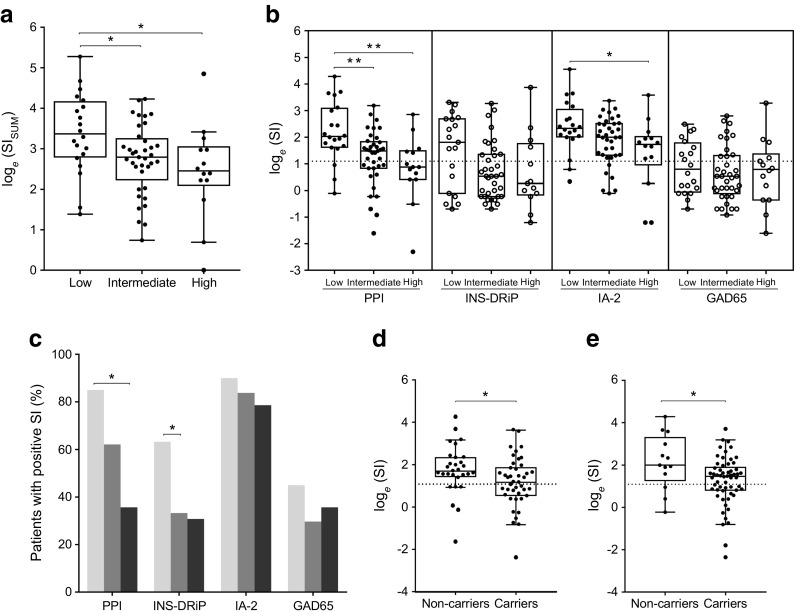
Sphingolipid-related SNPs associate with cellular islet autoimmunity in individuals with type 1 diabetes. Proliferation of T cells specific for GAD65, PPI, IA-2 and INS-DRiP in PBMCs freshly isolated from individuals with type 1 diabetes. Patients were divided into risk groups based on sphingolipid-related genetic risk: low (GRS=0.11–0.14, *n*=20); intermediate (GRS=0.14–0.16, *n*=37) and high (GRS >0.16, *n*=14). Data were normalised by natural log-transformation. SI ≥3 is considered positive. Tukey boxplots are shown. (**a**) Cumulative proliferation of islet-specific T cells (SI_SUM_ =SI_GAD65_+SI_PPI_+SI_IA-2_+SI_INS-DRiP_) in patient-risk groups. (**b**) Proliferation of T cells in patient-risk groups plotted per islet-autoantigen. Dashed line indicates SI=3. (**c**) Percentage of patients within each risk group with positive T cell proliferation responses (SI ≥3) plotted per islet-autoantigen. Light grey bars, low GRS; medium grey bars, intermediate GRS; dark grey bars, high GRS. (**d**, **e**) Proliferation of PPI-specific T cells in heterozygous and homozygous carriers of the (**d**) rs12150079 or (**e**) rs33988101 risk allele vs non-carriers of the respective risk allele. *p<0.05; **p<0.01. One-way ANOVA with Tukey’s multiple comparisons, *χ*^2^ two-proportions *Z* test and two-tailed unpaired Student’s *t* test

When comparing the GRS with the proliferation against all islet autoantigens (SI_SUM_ = SI_GAD65_ + SI_PPI_ + SI_IA-2_ + SI_INS-DRiP_) we surprisingly found that islet-specific T cells from intermediate- and high-risk patients proliferated less than those with a low-risk (*p* = 0.047 and *p* = 0.017, respectively; Fig. 3a). Focusing on individual islet autoantigens, we found that PPI-specific T cells proliferated less in intermediate- and high-risk patients compared with those at low-risk (*p* = 0.007 and *p* = 0.002, respectively) and IA-2-specific T cells proliferated less in high-risk patients compared with those at low-risk (*p* = 0.018), with a non-significant difference in the intermediate group (*p* = 0.31); there were also non-significant differences in proliferation for INS-DRiP among both high- and intermediate risk groups compared with the low-risk group (*p* = 0.159 and *p* = 0.069, respectively), but not for GAD65 (Fig. 3b). Looking at the percentage of patients in each risk group with positive proliferation responses against individual islet autoantigens (SI ≥3), we found that fewer patients in the high-risk group responded to PPI compared with the low-risk group (*p* = 0.003; Fig. 3c). Moreover, even though there were no significant differences in absolute INS-DRiP-specific T cell proliferation, the percentage of patients with a positive INS-DRiP response was significantly lower in the intermediate-risk group than in the low-risk group (*p* = 0.034). Furthermore, we found that heterozygous and homozygous carriers of the risk alleles rs12150079 (*ORMDL3*) and rs33988101 (*SPHK2*) had lower levels of T cell autoimmunity (*p* = 0.042 and *p* = 0.017, respectively; Fig. 3d,e).

### Increasing sulfatide levels in mice pancreatic islets is associated with prevention of autoimmune diabetes in NOD mice

**Fig. 4:**
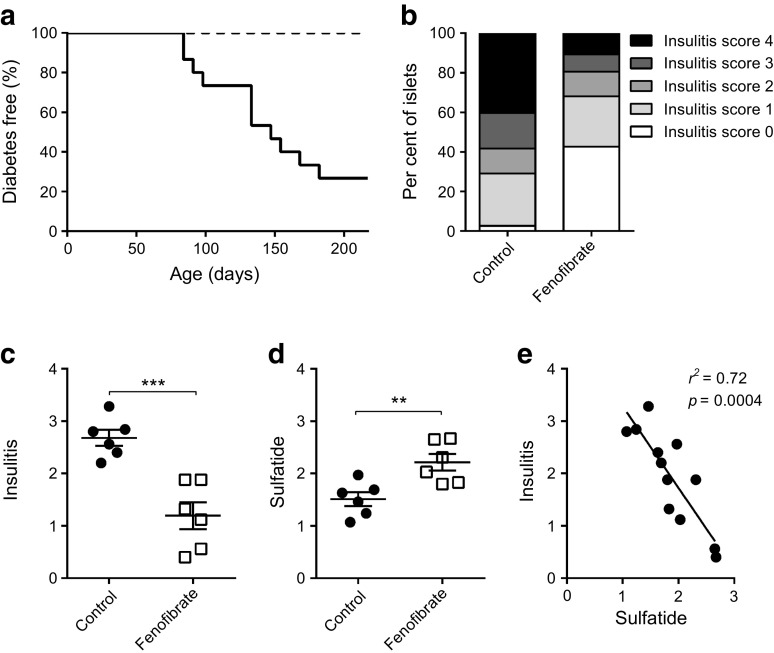
Fenofibrate prevents diabetes in NOD mice. NOD mice were treated with fenofibrate or control from an age of 3 weeks. (**a**) Diabetes incidence in the experimental groups (*n*=15); dotted line, fenofibrate; solid line, control. (**b**) Percentage distribution of insulitis. Insulitis score (*n*=6 per group) at age 13 weeks on a scale from 0 (no insulitis) to 4 (above 50% infiltration). (**c**) Average insulitis score. (**d**) Sulfatide score (*n*=6 per group). (**e**) Correlation between insulitis score and sulfatide with linear regression. Show is mean ± SEM. **p<0.01; ***p<0.001. Logrank Mantel–Cox, two-tailed unpaired Student’s *t* test and linear regression

Based on these findings, we considered pharmacological upregulation of pancreatic sulfatide levels as a possible therapeutic approach in type 1 diabetes. NOD mice were therefore treated with fenofibrate, which is known to increase sulfatide levels in several organs [37]. NOD mice, which usually develop insulitis at age 4 weeks [38], were treated with fenofibrate from an age of 3 weeks till 35 weeks. Development of diabetes was prevented in the fenofibrate treated mice (0/15 (0%) vs 11/15 (73%) in the control group (*p* < 0.0001; Fig. 4a). Fenofibrate also reduced the degree of insulitis (*p* = 0.0006; Fig. 4b,c) and increased the expression of sulfatide in islets (*p* = 0.007; Fig. 4d). There was an inverse correlation between sulfatide and insulitis score (*p* = 0.0004, *r*^2^ = 0.72; Fig. 4e) in the experimental animals. Fenofibrate treatment initiated after onset of diabetes reversed diabetes in 46% (6/13) NOD mice after 3 weeks of treatment.

## Discussion

**Fig. 5:**
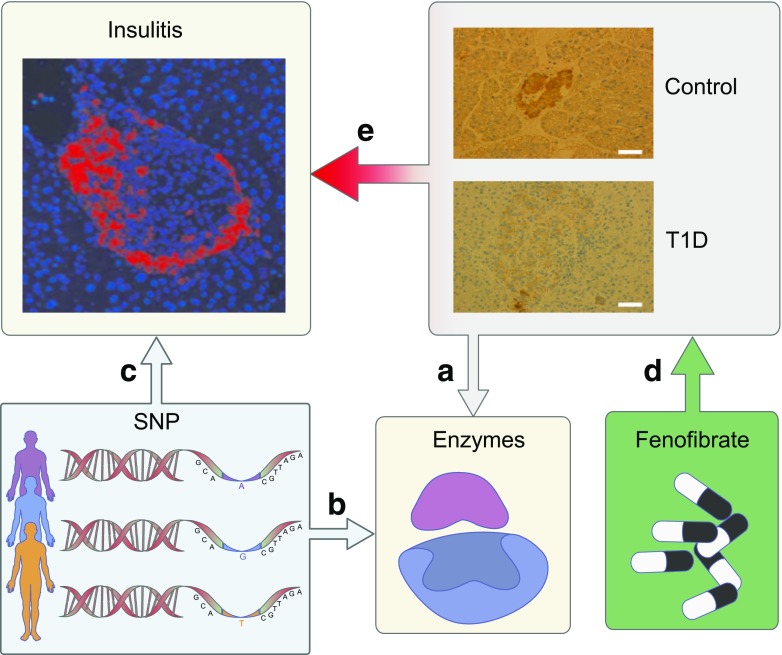
Sphingolipid metabolism is connected with type 1 diabetes**.** Overview of how sphingolipid metabolism is related to the development of type 1 diabetes. (**a**) There is a reduced amount of sulfatide in islets of individuals with newly diagnosed type 1 diabetes (images taken from Fig. 1b). This is related to altered expression of enzymes involved in the biosynthesis of sphingolipids in islets. (**b**) Genetic polymorphisms in the promoter region of eight genes encoding enzymes involved in sphingolipid metabolism increases the risk for developing type 1 diabetes. (**c**) These genetic polymorphisms are associated with lower rates of T cell proliferation when presented to beta cell autoantigens. (**d**) Fenofibrate stimulates sulfatide production in islets of NOD mice. (**e**) This is associated with complete protection against diabetes and a lower degree of insulitis in NOD mice; insulitis image adapted from [50] (original courtesy of A. van Halteren) with permission of Springer Nature

The present study provides human data demonstrating that sphingolipid metabolism contributes to genetic disease predisposition and autoimmunity and that the onset of type 1 diabetes is associated with altered sphingolipid metabolism and reduced expression of sulfatide in pancreatic islets (Fig. 5). Sulfatide is known to participate in the regulation of first-phase insulin secretion [13] and it is possible that the observed loss of pancreatic sulfatide may contribute to the loss of first-phase insulin secretion seen during the development of type 1 diabetes [39]. We notice a finer granulation in the islets of individuals with type 1 diabetes; whether this might, in part, influence the reduced sulfatide-staining that is seen in insulin granules, is unknown [14]. Reduced levels of sulfatide might be explained by reduced enzyme expression as suggested by the transcriptome analysis, which showed reduced expression of several enzymes involved in sphingolipid metabolism in islets from individuals with newly diagnosed type 1 diabetes (Fig. 5a). The reduced expression of *CERS2* is particularly interesting since this points towards altered hydrocarbon chain lengths of beta cell sphingolipids in type 1 diabetes, with lower amounts of long chains [40]. Long chain (C24) sulfatide is protective against diabetes development in NOD mice [41]. Furthermore, a low-grade enteroviral infection was found in the beta cells of all DiViD participants [5] and this could be linked to lower expression of C24 sulfatide which stimulates the natural killer (NK) T cells that normally eliminate diabetogenic viruses [41, 42]. We did not find any changes in the expression of arylsulfatase A, which degrades sulfatide in the lysosome [43], suggesting that the reduced amount of sulfatide did not result from enhanced rates of lipid degradation. The changed expression of *B3GALT5*, *B3GALT4* and *B4GALT1* suggest that the development of type 1 diabetes is associated with changes in the composition of islet glycosphingolipids with increases in the neolacto/lacto series and decreased levels of gangliosides (ESM Fig. 2). An altered amount of gangliosides could play a role in type 1 diabetes aetiology as ganglioside autoantibodies are found at the onset of disease [44]. Altered levels of ceramide and sphingosine-1-phosphate could also affect islet function through regulation of the sphingolipid rheostat [45].

To support these findings, GWAS data were interrogated and SNPs in the promoter regions of eight genes influencing sphingolipid levels were identified (Fig. 5b). Among these, the OR of 1.47 calculated for *B4GALNT1* ranks it among the highest risk genes implicated in the predisposition to type 1 diabetes (ESM Fig. 3). All SNPs identified here correlated with predisposition to type 1 diabetes but not type 2 diabetes. It should be noted that the SNPs identified could be inhibitory or activating and so the overall effect of these SNPs on sphingolipid composition is difficult to predict.

The most promising SNPs were found to be associated with lower rates of T cell proliferation when these cells were presented with beta cell autoantigens (Fig. 5c). This effect was linked with autoimmunity to PPI and to a lesser degree IA-2 and INS-DRiP, but not GAD65. A possible explanation for this seemingly paradoxical finding is that sulfatide is involved in PPI folding [46] and likely the formation of INS-DRiP. T cells recognise folded PPI and so impaired folding due to less sulfatide would lead to a lower immune response against PPI. A lack of sulfatide on the other hand would not affect autoimmunity to GAD65.

Fenofibrate has been in use for decades to reduce LDL-cholesterol, triacylglycerol and cholesterol levels and has shown beneficial effects on the prevention of diabetic neuropathy and retinopathy [47]. Here we demonstrate that NOD mice were protected from insulitis and diabetes by early exposure to fenofibrate and that this correlated with an increase in islet sulfatide levels (Fig. 5d,e). The positive effect of fenofibrate, however, cannot be solely credited to the increased amount of sulfatide as fenofibrate is also likely to affect other aspects of lipid biology. Previous studies have otherwise shown that the ceramide synthase inhibitor FTY720 [48] prevented diabetes development in NOD mice [49], highlighting the diverse roles of different sphingolipids in diabetes pathology.

In conclusion, we provide human evidence of an altered islet sphingolipid metabolism in type 1 diabetes*.* Increasing sulfatide levels prevents diabetes in NOD mice suggesting that upregulation of sulfatide biosynthesis may represent a promising therapeutic route in type 1 diabetes.

## Electronic supplementary material

ESM (PDF 480 kb)

## Data Availability

The RNA expression data is available online at https://www.dropbox.com/s/93mk5tzl5fdyo6b/Abnormal%20islet%20sphingolipid%20metabolism%20in%20type%201%20diabetes%2C%20RNA%20expression.xlsx?dl=0 A list of SNPs identified is available at https://www.dropbox.com/s/yfojma9xanpp2ju/Abnormal%20islet%20sphingolipid%20metabolism%20in%20type%201%20diabetes%20SNP.xlsx?dl=0

## Abbreviations

CPM: Counts per min
DiViD: Diabetes virus detection
eQTL: Expression quantitative trait loci
GRS: Genetic risk score
IA-2: Islet antigen-2
INS-DRiP: Insulin-defective ribosomal product
PBMC: Peripheral blood mononuclear cell
PPI: Preproinsulin
SI: Stimulation index

## References

[1] van Belle TL, Coppieters KT, von Herrath MG (2011). Type 1 diabetes: etiology, immunology, and therapeutic strategies. Physiol Rev.

[2] Leete P, Willcox A, Krogvold L (2016). Differential insulitic profiles determine the extent of beta-cell destruction and the age at onset of type 1 diabetes. Diabetes.

[3] Krogvold L, Wiberg A, Edwin B (2016). Insulitis and characterisation of infiltrating T cells in surgical pancreatic tail resections from patients at onset of type 1 diabetes. Diabetologia.

[4] Coppieters KT, Dotta F, Amirian N (2012). Demonstration of islet-autoreactive CD8 T cells in insulitic lesions from recent onset and long-term type 1 diabetes patients. J Exp Med.

[5] Krogvold L, Skog O, Sundstrom G (2015). Function of isolated pancreatic islets from patients at onset of type 1 diabetes: insulin secretion can be restored after some days in a nondiabetogenic environment in vitro: results from the DiViD study. Diabetes.

[6] Malmegrim KC, de Azevedo JT, Arruda LC (2017). Immunological balance is associated with clinical outcome after autologous hematopoietic stem cell transplantation in type 1 diabetes. Front Immunol.

[7] Maceyka M, Spiegel S (2014). Sphingolipid metabolites in inflammatory disease. Nature.

[8] Chen Y, Liu Y, Sullards MC, Merrill AH (2010). An introduction to sphingolipid metabolism and analysis by new technologies. NeuroMolecular Med.

[9] Yamaji T, Hanada K (2015). Sphingolipid metabolism and interorganellar transport: localization of sphingolipid enzymes and lipid transfer proteins. Traffic.

[10] Boslem E, Meikle PJ, Biden TJ (2012). Roles of ceramide and sphingolipids in pancreatic beta-cell function and dysfunction. Islets.

[11] Veret J, Bellini L, Giussani P, Ng C, Magnan C, Le Stunff H (2014). Roles of sphingolipid metabolism in pancreatic beta cell dysfunction induced by lipotoxicity. J Clin Med.

[12] Ng ML, Wadham C, Sukocheva OA (2017). The role of sphingolipid signalling in diabetesassociated pathologies (Review). Int J Mol Med.

[13] Buschard K, Blomqvist M, Mansson JE, Fredman P, Juhl K, Gromada J (2006). C16:0 sulfatide inhibits insulin secretion in rat beta-cells by reducing the sensitivity of KATP channels to ATP inhibition. Diabetes.

[14] Buschard K, Bracey AW, McElroy DL (2016). Sulfatide preserves insulin crystals not by being integrated in the lattice but by stabilizing their surface. J Diabetes Res.

[15] Krogvold L, Edwin B, Buanes T (2014). Pancreatic biopsy by minimal tail resection in live adult patients at the onset of type 1 diabetes: experiences from the DiViD study. Diabetologia.

[16] Osterbye T, Funda DP, Fundova P, Mansson JE, Tlaskalova-Hogenova H, Buschard K (2010). A subset of human pancreatic beta cells express functional CD14 receptors: a signaling pathway for beta cell-related glycolipids, sulfatide and beta-galactosylceramide. Diabetes Metab Res Rev.

[17] Fredman P, Mattsson L, Andersson K (1988). Characterization of the binding epitope of a monoclonal antibody to sulphatide. Biochem J.

[18] Campbell-Thompson M, Wasserfall C, Kaddis J (2012). Network for Pancreatic Organ Donors with Diabetes (nPOD): developing a tissue biobank for type 1 diabetes. Diabetes Metab Res Rev.

[19] Richardson SJ, Rodriguez-Calvo T, Gerling IC (2016). Islet cell hyperexpression of HLA class I antigens: a defining feature in type 1 diabetes. Diabetologia.

[20] Wu J, Kakoola DN, Lenchik NI, Desiderio DM, Marshall DR, Gerling IC (2012). Molecular phenotyping of immune cells from young NOD mice reveals abnormal metabolic pathways in the early induction phase of autoimmune diabetes. PLoS One.

[21] Onengut-Gumuscu S, Chen WM, Burren O (2015). Fine mapping of type 1 diabetes susceptibility loci and evidence for colocalization of causal variants with lymphoid gene enhancers. Nat Genet.

[22] Rosenbloom KR, Sloan CA, Malladi VS (2013). ENCODE data in the UCSC Genome Browser: year 5 update. Nucleic Acids Res.

[23] Boyle AP, Hong EL, Hariharan M (2012). Annotation of functional variation in personal genomes using RegulomeDB. Genome Res.

[24] Consortium G (2015). Human genomics. The Genotype-Tissue Expression (GTEx) pilot analysis: multitissue gene regulation in humans. Science.

[25] Stranger BE, Montgomery SB, Dimas AS (2012). Patterns of cis regulatory variation in diverse human populations. PLoS Genet.

[26] Westra HJ, Peters MJ, Esko T (2013). Systematic identification of trans eQTLs as putative drivers of known disease associations. Nat Genet.

[27] Franken KL, Hiemstra HS, van Meijgaarden KE (2000). Purification of his-tagged proteins by immobilized chelate affinity chromatography: the benefits from the use of organic solvent. Protein Expr Purif.

[28] Kracht MJ, van Lummel M, Nikolic T (2017). Autoimmunity against a defective ribosomal insulin gene product in type 1 diabetes. Nat Med.

[29] Hanada K (2003). Serine palmitoyltransferase, a key enzyme of sphingolipid metabolism. Biochim Biophys Acta.

[30] Siow D, Sunkara M, Dunn TM, Morris AJ, Wattenberg B (2015). ORMDL/serine palmitoyltransferase stoichiometry determines effects of ORMDL3 expression on sphingolipid biosynthesis. J Lipid Res.

[31] Han G, Gupta SD, Gable K (2009). Identification of small subunits of mammalian serine palmitoyltransferase that confer distinct acyl-CoA substrate specificities. Proc Natl Acad Sci U S A.

[32] El-Hattab AW (2016). Serine biosynthesis and transport defects. Mol Genet Metab.

[33] Floyel T, Kaur S, Pociot F (2015). Genes affecting beta-cell function in type 1 diabetes. Curr Diab Rep.

[34] Mirza AH, Kaur S, Brorsson CA, Pociot F (2014). Effects of GWAS-associated genetic variants on lncRNAs within IBD and T1D candidate loci. PLoS One.

[35] Baranowski M, Gorski J (2011). Heart sphingolipids in health and disease. Adv Exp Med Biol.

[36] Barrett JC, Clayton DG, Concannon P (2009). Genome-wide association study and meta-analysis find that over 40 loci affect risk of type 1 diabetes. Nat Genet.

[37] Nakajima T, Kamijo Y, Yuzhe H (2013). Peroxisome proliferator-activated receptor alpha mediates enhancement of gene expression of cerebroside sulfotransferase in several murine organs. Glycoconj J.

[38] Crevecoeur I, Gudmundsdottir V, Vig S (2017). Early differences in islets from prediabetic NOD mice: combined microarray and proteomic analysis. Diabetologia.

[39] Sosenko JM, Skyler JS, Beam CA (2013). Acceleration of the loss of the first-phase insulin response during the progression to type 1 diabetes in diabetes prevention trial-type 1 participants. Diabetes.

[40] Laviad EL, Albee L, Pankova-Kholmyansky I (2008). Characterization of ceramide synthase 2: tissue distribution, substrate specificity, and inhibition by sphingosine 1-phosphate. J Biol Chem.

[41] Subramanian L, Blumenfeld H, Tohn R (2012). NKT cells stimulated by long fatty acyl chain sulfatides significantly reduce the incidence of type 1 diabetes in nonobese diabetic mice [corrected]. PLoS One.

[42] Exley MA, Bigley NJ, Cheng O (2001). CD1d-reactive T cell activation leads to amelioration of disease caused by diabetogenic encephalomyocarditis virus. J Leukoc Biol.

[43] Doerr J, Bockenhoff A, Ewald B (2015). Arylsulfatase A overexpressing human iPSC-derived neural cells reduce CNS sulfatide storage in a mouse model of metachromatic leukodystrophy. Mol Ther.

[44] Dotta F, Falorni A, Tiberti C (1997). Autoantibodies to the GM2-1 islet ganglioside and to GAD-65 at type 1 diabetes onset. J Autoimmun.

[45] Jessup CF, Bonder CS, Pitson SM, Coates PT (2011). The sphingolipid rheostat: a potential target for improving pancreatic islet survival and function. Endocr Metab Immune Disord Drug Targets.

[46] Osterbye T, Jorgensen KH, Fredman P (2001). Sulfatide promotes the folding of proinsulin, preserves insulin crystals, and mediates its monomerization. Glycobiology.

[47] Wright AD, Dodson PM (2011). Medical management of diabetic retinopathy: fenofibrate and ACCORD Eye studies. Eye (Lond).

[48] Berdyshev EV, Gorshkova I, Skobeleva A (2009). FTY720 inhibits ceramide synthases and up-regulates dihydrosphingosine 1-phosphate formation in human lung endothelial cells. J Biol Chem.

[49] Yang Z, Chen M, Fialkow LB (2003). The immune modulator FYT720 prevents autoimmune diabetes in nonobese diabetic mice. Clin Immunol.

[50] Roep BO (2003). The role of T-cells in the pathogenesis of type 1 diabetes: from cause to cure. Diabetologia.

