# Supplementary material for: Abnormal islet sphingolipid metabolism in type 1 diabetes
Source: Diabetologia. 2018 Apr 18;61(7):1650–61. doi: 10.1007/s00125-018-4614-2 (PMC6445476; doi:10.1007/s00125-018-4614-2)
Supplement: Supplementary file 1 — (PDF 480 kb) [file 125_2018_4614_MOESM1_ESM.pdf]

Electronic Supplementary Material

ESM Table 1 List of human samples **(a)** Tissue samples. **(b)** Samples used for transcriptome analysis. **(c)** Samples used for T cell proliferation.

**a) Tissue samples**					
Patient	Age (years)	Sex	Disease	Disease duration	Source
DiViD 6	35	Male	T1D	5w	DiViD
DiViD 5	24	Female	T1D	5w	DiViD
DiViD 4	31	Male	T1D	5w	DiViD
DiViD 3	34	Female	T1D	9w	DiViD
DiViD 2	24	Male	T1D	3w	DiViD
DiViD 1	25	Female	T1D	4w	DiViD
28 74	52	Female	No diabetes	N/A	Exeter
540 91	11	Male	No diabetes	N/A	Exeter
8579	7	UNK	No diabetes	N/A	Exeter
E560	17	Male	T1D	1w	Exeter
E124B	42	Female	T1D	18m	Exeter
11746	6	Male	T1D	<1w	Exeter
1	Unknown	Unknown	No diabetes	N/A	Rigshospitalet
2	Unknown	Unknown	No diabetes	N/A	Rigshospitalet
3	Unknown	Unknown	No diabetes	N/A	Rigshospitalet
**b) Transcriptome analysis**					
Patient	Age (years)	Sex	Disease	Disease duration	Source
6013-04PT	65	Male	No diabetes	N/A	nPOD
6024-01PT	21	Male	No diabetes	N/A	nPOD

6048-04PT	30	Male	No diabetes	N/A	nPOD
6075-06PB	16	Male	No diabetes	N/A	nPOD
6012-02PT	68	Female	No diabetes	N/A	nPOD
6099-02PB	14	Male	No diabetes	N/A	nPOD
6140-04PT	38	Male	No diabetes	N/A	nPOD
6162-01PT	22	Male	No diabetes	N/A	nPOD
6168-04PT	51	Male	No diabetes	N/A	nPOD
6227-02PT	17	Female	No diabetes	N/A	nPOD
6129-02PT	42.9	Female	No diabetes	N/A	nPOD
6165-04PT	45.8	Female	No diabetes	N/A	nPOD
6102-04PT	45.1	Female	No diabetes	N/A	nPOD
6229-02PT	31	Female	No diabetes	N/A	nPOD
6251-02PT	33	Female	No diabetes	N/A	nPOD
6010-04PT	47	Female	No diabetes	N/A	nPOD
6179-04B PB	21.8	Female	No diabetes	N/A	nPOD
6019-04PT	42	Male	No diabetes	N/A	nPOD
6188-06PT	36.1	Male	T2D	Unknown	nPOD
6114-03PT	42.8	Male	T2D	Unknown	nPOD
6249-01PT	45	Female	T2D	Unknown	nPOD
6275-02PT	48	Male	T2D	Unknown	nPOD
6273-04PT	45	Female	T2D	Unknown	nPOD
6191-01PT	62	Female	T2D	Unknown	nPOD
6059-06PT	18.8	Female	T2D	Unknown	nPOD
6110-05PT	20.7	Female	T2D	Unknown	nPOD
DiViD 6	35	Male	T1D	5w	DiViD
DiViD 5	24	Female	T1D	5w	DiViD
DiViD 4	31	Male	T1D	5w	DiViD

DiViD 3	34	Female	T1D	9w	DiViD
DiViD 2	24	Male	T1D	3w	DiViD
**d) T cell proliferation**					
Patient	Age (years)	Sex	Disease	Disease duration (years)	GRS group
55	19.6	Female	T1D	8.2	HIGH
57	1.3	Male	T1D	0.3	INTERMEDIATE
65	18.7	Male	T1D	16.8	INTERMEDIATE
66	13.9	Male	T1D	8.0	INTERMEDIATE
68	19.0	Male	T1D	4.5	INTERMEDIATE
76	20.1	Male	T1D	16.7	LOW
77	20.1	Male	T1D	8.1	LOW
79	16.2	Female	T1D	3.8	INTERMEDIATE
80	16.1	Female	T1D	11.0	HIGH
81	13.1	Female	T1D	6.7	INTERMEDIATE
83	18.0	Male	T1D	8.3	INTERMEDIATE
84	16.9	Female	T1D	8.2	INTERMEDIATE
85	39.8	Male	T1D	32.0	INTERMEDIATE
86	12.3	Female	T1D	0.8	INTERMEDIATE
87	11.1	Male	T1D	2.0	LOW
88	23.1	Female	T1D	21.8	LOW
89	23.8	Male	T1D	12.0	INTERMEDIATE
90	24.3	Female	T1D	13.9	LOW
91	23.8	Female	T1D	21.6	INTERMEDIATE
92	13.7	Male	T1D	3.9	INTERMEDIATE
93	18.4	Male	T1D	6.6	HIGH
94	13.8	Male	T1D	0.0	HIGH

95	21.6	Male	T1D	6.3	INTERMEDIATE
96	17.9	Female	T1D	16.2	HIGH
97	11.2	Female	T1D	0.0	INTERMEDIATE
98	13.4	Male	T1D	0.0	LOW
100	14.0	Male	T1D	12.1	HIGH
101	11.7	Female	T1D	0.1	INTERMEDIATE
103	13.6	Male	T1D	0.1	INTERMEDIATE
104	19.5	Male	T1D	4.6	INTERMEDIATE
105	13.3	Female	T1D	0.0	HIGH
106	18.7	Male	T1D	13.0	INTERMEDIATE
107	19.9	Male	T1D	14.3	LOW
108	10.4	Female	T1D	0.0	HIGH
109	5.9	Male	T1D	0.0	HIGH
110	16.4	Female	T1D	11.2	INTERMEDIATE
111	19.5	Female	T1D	11.6	INTERMEDIATE
112	18.8	Male	T1D	16.2	LOW
113	16.7	Male	T1D	6.0	INTERMEDIATE
114	17.2	Male	T1D	0.2	HIGH
115	22.6	Female	T1D	12.3	HIGH
125	15.4	Male	T1D	0.0	INTERMEDIATE
127	11.4	Female	T1D	8.4	INTERMEDIATE
129	17.1	Female	T1D	10.1	LOW
134	20.7	Male	T1D	10.2	HIGH
135	13.6	Male	T1D	1.5	INTERMEDIATE
136	20.8	Female	T1D	11.5	LOW
137	21.4	Male	T1D	19.2	LOW
138	21.3	Male	T1D	Unknown	INTERMEDIATE

139	19.3	Female	T1D	6.1	INTERMEDIATE
140	18.8	Male	T1D	Unknown	LOW
141	14.2	Male	T1D	8.3	LOW
142	14.5	Male	T1D	6.8	LOW
143	29.1	Female	T1D	5.7	LOW
144	34.3	Female	T1D	25.4	LOW
145	34.9	Female	T1D	0.0	LOW
147	18.0	Male	T1D	3.0	INTERMEDIATE
148	8.8	Female	T1D	0.0	INTERMEDIATE
149	23.9	Male	T1D	12.5	INTERMEDIATE
150	18.1	Male	T1D	5.9	LOW
152	14.6	Male	T1D	1.1	INTERMEDIATE
153	18.3	Female	T1D	Unknown	INTERMEDIATE
154	23.5	Male	T1D	20.8	INTERMEDIATE
155	21.3	Female	T1D	12.9	INTERMEDIATE
156	29.6	Female	T1D	Unknown	LOW
157	14.9	Male	T1D	7.9	INTERMEDIATE
159	6.6	Male	T1D	0.1	LOW
160	20.6	Female	T1D	16.8	HIGH
161	10.3	Female	T1D	0.1	INTERMEDIATE
162	18.9	Male	T1D	8.4	INTERMEDIATE
163	13.7	Female	T1D	0.0	HIGH

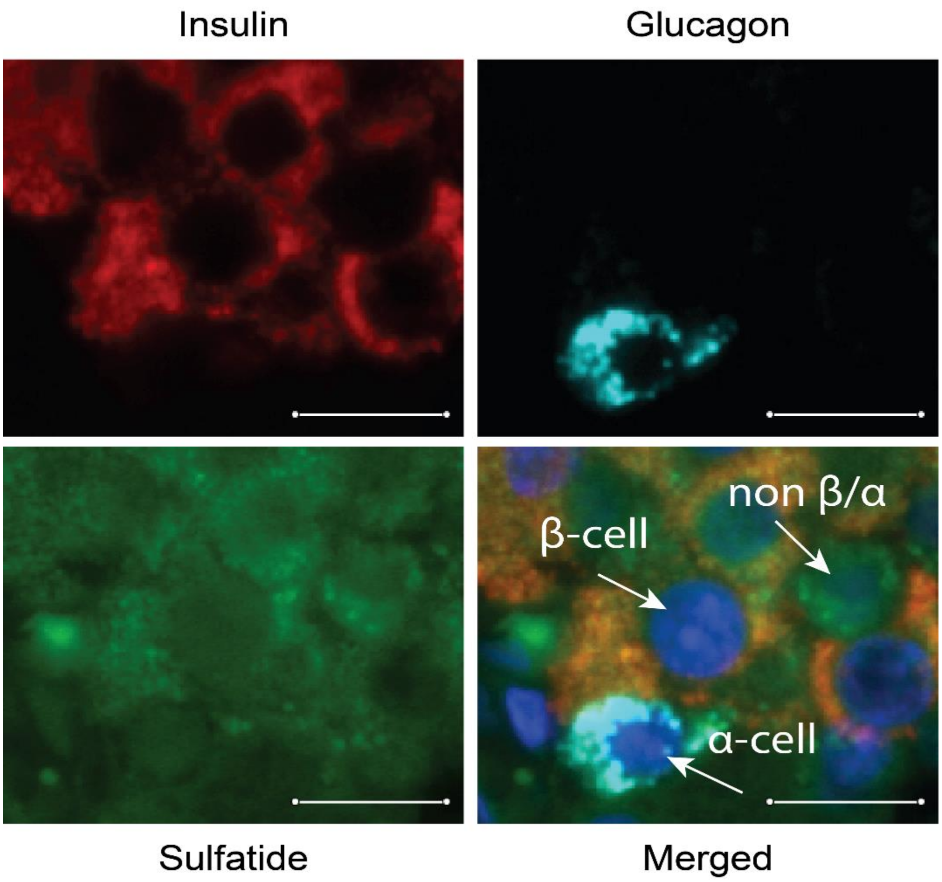

ESM Fig. 1 Sulfatide is present in beta cells and not alpha cells. Immunofluorescent staining of a healthy pancreas sections showing that sulfatide is expressed in beta cells and occasionally in cells negative for both insulin and glucagon. Scale bar, 10 µm.

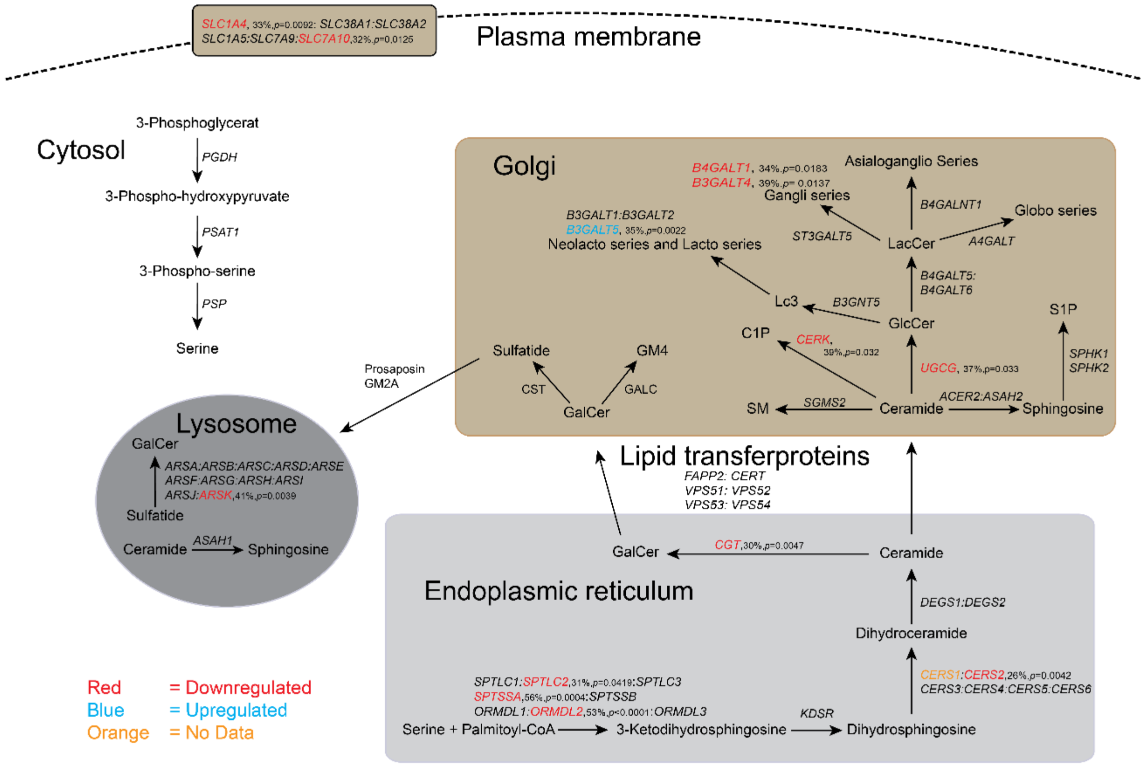

ESM Fig. 2 Overview of the altered RNA expression in pancreatic islets of individuals with newly diagnosed type 1 diabetes vs control participants**.** The enzymes are listed next to the catalytic reaction they are involved in. It should be noted that some enzymes catalyze more reactions than shown here. The number after the enzymes name represents the change in percent followed by the *p* value. The difference in expression is showed for genes with a *p* value <0.05. No difference between control participants and individuals with type 2 diabetes for the selected genes. One-way ANOVA with Tukey's multiple comparisons test.

3-ketodihydrosphingosine reductase (*KDSR*); Δ4-dihydroceramide desaturase (*DEGS*); Galactosylceramide (GalCer); N-acylsphingosine amidohydrolase (*ASAH*); sphingomyelin (SM); C1P, ceramide-1-phosphate; S1P, sphingosine-1-phosphate; alkaline ceramidase (*ACER*); sphingomyelin synthase (*SGMS*); sphingosine kinase (*SPHK*)*:* cerebroside sulfotransferase (*CST*); galactosylceramidase (*GALC*); glucosylceramide (GlcCer); lactosylceramide (LacCer); pleckstrin homology domain containing A8 (*FAPP2* or *PLEKHA8);* ceramide transfer protein (*CERT);* VPS, GARP, complex subunit (*VPS).* solute

carrier family (*SLC*); GM2 ganglioside activator (*GM2A*); beta-1,3-galactosyltransferase (*B3GALT*); beta-1,4-galactosyltransferase (*B4GALT*); lactotriaosylceramide (Lc3); UDP-GlcNAc:betaGal beta-1,3-N-acetylglucosaminyltransferase (*B3GNT*); alpha 1,4-galactosyltransferase (*A4GALT*); beta-1,4-N-acetyl-galactosaminyltransferase (*B4GALNT*); galactose-α2,3-sialyltransferase (*ST3GALT*); phosphoglycerate dehydrogenase (*PGDH*); phosphoserine aminotransferase (*PSAT*); phosphoserine phosphatase (*PSP*)

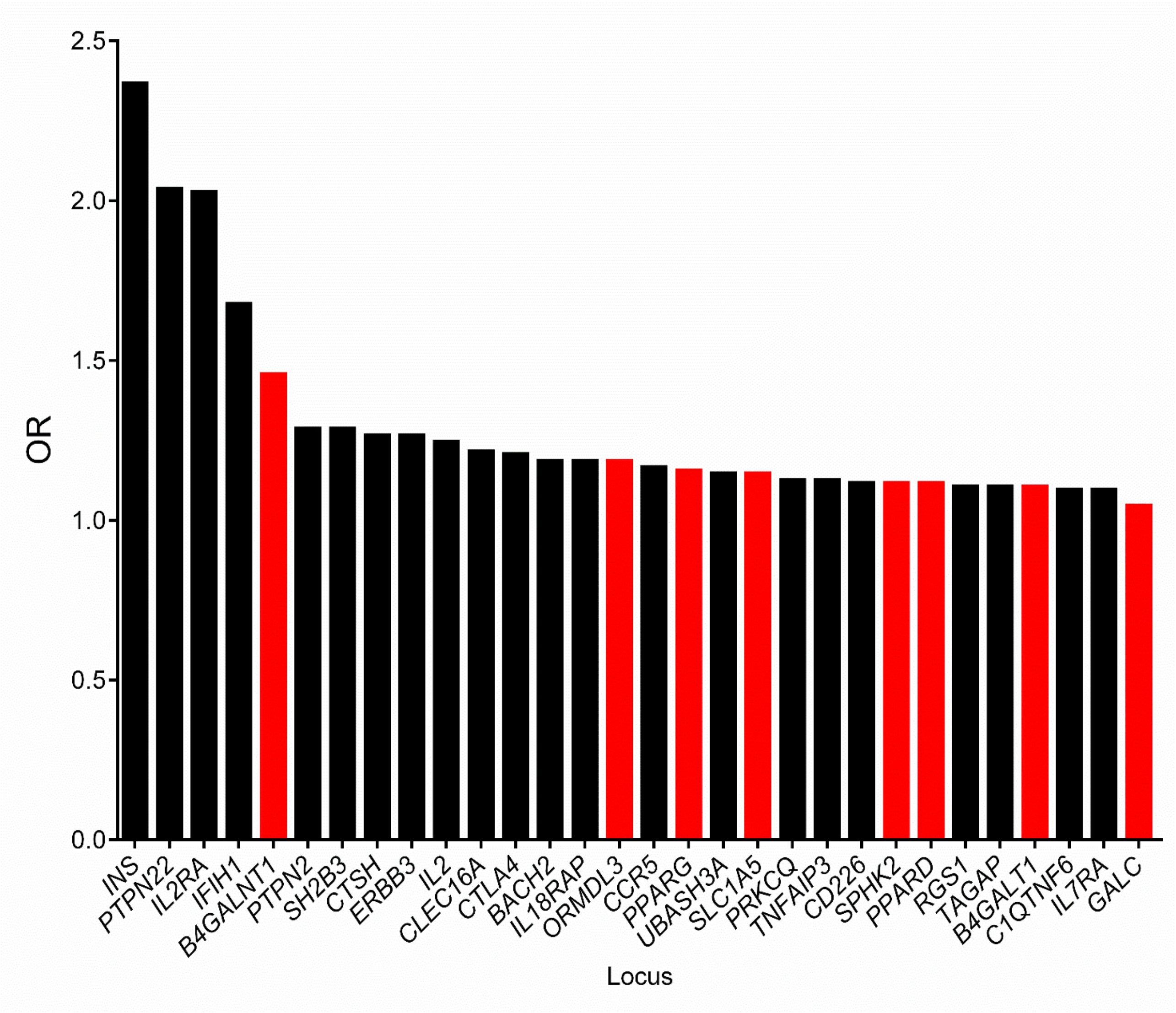

ESM Fig. 3 Ranked non-HLA loci associated with type 1 diabetes. Red bars represent genes identified in this study. List of genes taken from [1].

[1] Concannon P, Rich SS, Nepom GT (2009) Genetics of type 1A diabetes. N Engl J Med 360: 1646-1654
